# Effect of Laparoscopic Ovarian Drilling on Outcomes of *In Vitro* Fertilization in Clomiphene-Resistant Women with Polycystic Ovary Syndrome 

**DOI:** 10.22074/ijfs.2016.4767

**Published:** 2016-04-05

**Authors:** Maryam Eftekhar, Razieh Deghani Firoozabadi, Parisa Khani, Ehsan Ziaei Bideh, Hosein Forghani

**Affiliations:** 1 Research and Clinical Center for Infertility, Shahid Sadoughi University of Medical Sciences, Yazd, Iran; 2 Department of Health Education, Faculty of Health, Shahid Sadoughi University of Medical Sciences, Yazd, Iran

**Keywords:** Ovary, Surgical Diathermy, Polycystic Ovary Syndrome, IVF/ICSI, Assisted Reproductive Technology

## Abstract

**Background:**

Recently the laparoscopic ovarian drilling (LOD) has been used as a surgical treatment for ovulation in women with polycystic ovarian syndrome (PCOS), although its mechanism and outcomes are still unclear. This study was undertaken to evaluate the *in vitro* fertilization (IVF)/intracytoplasmic sperm injection (ICSI) outcomes in clomiphene-resistant women with PCOS who were treated with LOD.

**Materials and Methods:**

In this retrospective study, we reviewed the medical records of 300 women between 20 to 35 years old with clomiphene-resistant PCOS who had an ovulatory infertility and who were nominated for IVF/ICSI. Based on their treatment history, they were located into the following two groups: group I (n=150) including PCOS women who had history of LOD at least 6 months to 3 years before IVF/ICSI, and group II (n=150) including PCOS patients without history of drilling. Both groups were treated with antagonist protocol in the assisted reproductive technology (ART) process. The duration of treatment cycles, number of oocytes and embryos obtained, chemical and clinical pregnancy rate, the number of embryos transferred, and presence of ovarian hyper stimulation syndrome (OHSS) were measured. To compare means and frequencies, Student’s t test, Mann-whitney and chi-square tests were used.

**Results:**

Our results showed that ovarian cauterization before IVF/ICSI in patients with PCOS reduced the risk of OHSS (P=0.025). Despite the same pregnancy rate in both groups (P=0.604), more obtained oocytes and embryos were seen on women without ovarian drilling than women with LOD (P˂0.001 and P=0.033, respectively).

**Conclusion:**

There is no difference between the pregnancy rate in both groups. Due to significant reduction in OHSS in women undergoing LOD, this surgical treatment may be considered as a useful technique in the management of patients who have previously developed OHSS. However, there are ongoing concerns about long-term effects of LOD on ovarian function.

## Introduction

Polycystic ovary syndrome (PCOS) with 5-10% prevalence among the reproductive-age women involves reproductive and metabolic systems (1,3) that leads to ovulation dysfunction (4). Lifestyle modifications and administration of selective estrogen receptor modulators (SERMs), including clomiphene citrate (CC), are considered as the first-step approach in treatment for PCOS patients (2). But in 20% of cases, CC is not successful in ovulation induction (5). Gonadotropin therapy as the second option for these patients often causes overproduction of follicles that lead to risks of ovarian hyperstimulation syndrome (OHSS) and multiple pregnancies (6). Recently laparoscopic ovarian drilling (LOD) has been used widely by gynecologists as an alternative surgical method for ovulation induction using gonadotropins for PCOS patients unresponsive to clomiphene, but there is a lack of consensus on effectiveness of this method (7). In a study by Flyckt and Goldberg (8), they showed that serum luteinizing hormone (LH) and testosterone levels were normalized following LOD, while their levels remained unchanged over long-term follow-up. They also evaluated ovulation and pregnancy rates after gonadotropin therapy for ovulation induction and LOD. They concluded that although the mechanism of LOD is unknown, this method prevents the risks of multiple pregnancy and OHSS. Also several studies have reported the impact of LOD prior to assisted reproduction technology (ART) in decreasing the OHSS risk and improving the pregnancy rate in women with a history of cancellation of *in vitro* fertilization (IVF) treatment cycle due to risk of OHSS or even risk of OHSS in previous treatment cycle (9,11). Other study also showed that LOD can reduce the risk of cancellation of the ART treatment cycle, but there are no significant differences in pregnancy, miscarriage, or live birth rate (12). 

The effect of LOD on ART outcomes in clomipheneresistant PCOS patients is still unknown; therefore, we aimed to evaluate IVF/intracytoplasmic sperm injection (ICSI) outcomes in clomiphene-resistant women with PCOS who were treated with LOD. 

## Materials and Methods

After Institutional Review Board approval was received, a retrospective review of hospital records was performed at the Research and Clinical Center for Infertility, Yazd, Iran. In this study, about 1000 medical records of clomiphene-resistant PCOS women undergoing IVF/ICSI treatment from 2006 to 2010 were reviewed. The inclusion criteria were as follows: age between 20 and 35 years old, history of at least one year infertility, and no response to CC (dose up to 150 mg/day for at least three cycles) (13). Women with any other cause of oligomenorrhea and hyperandrogenism were excluded. Furthermore the patients with the following criteria were excluded: history of previous IVF/ICSI, chronic diseases such as thyroid disorders and diabetes mellitus, infertility due to severe male factor (azoospermia), severe endometriosis, and body mass index (BMI)>30. Therefore, 150 clomipheneresistant PCOS women meeting our inclusion criteria with a history of LOD, (performed at least 6 months to 3 years before IVF/ICSI) were assigned to the group I, while 150 clomiphene-resistant PCOS women with no history of electro-cauterization who underwent IVF/ ICSI were assighned to the group II (control group). Two groups were matched in terms of age, duration of infertility, and BMI. PCOS diagnosis was defined as having at least 2 signs of the following Rotterdam criteria: anovulation or oligomenorrhoea, clinical or biochemical signs of hyperandrogenism and the typical ultrasound (US) patterns (polycystic ovaries) (4). 

This study was approved by the Ethics Committee of Shahid Sadoughi University of Medical Sciences, Yazd, Iran, for collecting the data from medical records. 

Laparoscopy was performed under general anaesthesia, using 10 mm laparoscope and a unipolar needle electrode with a coagulating current set at 40 W power. In each ovary, four drilling points were made and duration of each diathermy application was about 3-5 seconds. 

The patients of groups I and II were treated with gonadotropin-releasing hormone (GnRH) antagonist protocol. They received 150 IU/daily of recombinant human follicle stimulating hormone (r-hFSH, Gonalf, Serono, Switzerland) from second day of menstrual cycle that was assessed by serial vaginal sonography. 

When mean diameter of dominant follicles reached to 14 mm, 0.25 mg/day of GnRH antagonist (Cetrotide, Sereno) was started and continued until the day of human chorionic gonadotropin (hCG, Pregnyl, Organon, Netherland) injection. When at least two follicles with a mean diameter of 17 mm or one leading follicle larger than 18 mm was observed, 10000 IU hCG was injected. Oocyte retrieval was done using a 17-gauge needle under vaginal ultrasonography guidance, 34-36 hours after hCG injection. Subsequently conventional IVF or ICSI was performed. 

In all patients, 2-3 embryos were transferred 2 days after oocyte retrieval using an embryo transfer catheter (Labotect Labor-Technik-Göttingen GmbH, Germany). The patients then inserted 800 mg daily Cyclogest suppository (Aburaihan, Iran) on the day of oocyte collection for luteal phase support, and it continued until the fetal heart activity was documented by ultrasonography. To determine chemical pregnancy, the serum hCG level on day 16 after the oocyte recovery was measured. Chemical pregnancy was defined by serum beta-hCG (β-hCG)>50 IU/L, and clinical pregnancy was defined by observation of fetal heart activity by transvaginal ultrasonography 2-3 weeks after positive β-hCG. 

The patients were considered at risk of OHSS if more than 15 follicles over 14 mm were observed in each ovary and serum estradiol (E2) levels were more than 3000 pg/ml on the day of hCG administration. In these patients, cycle was canceled; embryos were frozen and not transferred. 

The following outcome measures were compared between two groups: duration of treatment cycles, the number of oocytes obtained, chemical and clinical pregnancy rate, number of embryos obtained, the number of embryos transferred, and the risk of OHSS. 

## Statistical analysis

Data was analyzed using Statistical Package for the Social Sciences 16.0 (SPSS, SPSS Inc., USA). Normal quantitative variables were described as mean ± SD and 95% confidence interval (CI), qualitative data were presented as frequency, and categorical variables were expressed as a percentage. Student’s t test and Mann-Whitney U test were used to ascertain the significance of differences between mean values of the variables such as demographic characteristics, number of oocytes and embryo obtained. Chi-squares analysis (χ² tests) was performed to meausr the proportions of categorical variables between two groups. P value<0.05 was cosidered as statiscally significant. 

## Results

From 1000 medical records of clomiphene-resistant PCOS patients who underwent IVF/ICSI treatment and who were reffered to our center from 2006 to 2010, 300 women were enrolled in the study and assigned to two groups (n=150/each). 

The demographic, clinical and endocrinological characteristics of participants are showed in Table 1. There were no significant difference in mean age, BMI, duration and type of infertility, and duration of treatment between two groups, but basal FSH (day 3 FSH) levels in groups I and II showed a statisticaly significant difference (Table 1,P=0.019). 

There was no significant difference between two groups regarding chemical and clinical pregnancy rate (P=0.604), but mean number of oocytes and embryos obtained were more in group II (P=0.001); however, this difference was not clinically significant (Table 2). Among 150 patients who were treated by electro-cauterization, 10 women (6.7%) were diagnosed with OHSS as compared with 22 (7.14%) patients in group II, indicating that there is a significant difference (P=0.025, Table 2). 

**Table 1:** Demographic, clinical and endocrinological characteristics of participants in two groups


Characteristics	Group I	Group II	P value(Student t test)

Age (Y)*	27.96 ± 3.82	27.21 ± 4.13	0.106
BMI (kg/m2)*	25.02 ± 2.71	24.86 ± 2.55	0.569
Duration of infertility (Y)*	7.01 ± 2.52	6.64 ± 2.75	0.222
Basal FSH level (day 3 FSH) (IU/L)*	6.64 ± 1.83	5.93 ± 1.89	0.019**
Duration of treatment cycle (IVF/ICSI) (day)*	12.06 ± 1.18	11.88 ± 1.13	0.197
	n (%)	n (%)	P value (Chi-square test)
Type of infertility		
Primary	136(90.7%)	139(92.7%)	0.531
Secondary	14(9.3%)	11(7.3%)	


*; All data are presented as mean ± SD. **; Significant at P<0.05, BMI; Body mass index, IVF; In vitro fertilization, ICSI; Intracytoplasmic sperm injection and FSH; Follicle-stimulating hormone.

**Table 2:** ART outcomes in two groups


	With LOD	Without LOD	P value(Mann-Whitney test)

Number of oocytes obtained*	12.44 ± 3.25	13.48 ± 3.02	<0.001**
Number of embryo obtained*	9.84 ± 2.65	10.50 ± 2.67	0.033**
	n (%)	n (%)	P value (Chi-squqre test)
Chemical pregnancy	61 (40.7%)	60 (40%)	0.906
Clinical pregnancy	53 (35.3%)	52 (34.7%)	0.604
Chemical pregnancy	61 (40.7%)	60 (40%)	0.906
OHSS	10 (6.7%)	22 (14.7%)	0.025**


*; All data are presented as mean ± SD. **; Significant at P<0.05, OHSS; Ovarian hyperstimulation syndrome, ART; Assisted reproductive technology and LOD; Laparoscopic ovarian drilling.

## Discussion

As an alternative to treatment of clomipheneresistant patients with PCOS, LOD has been proposed due to its quick and easy approach (8). 

In this research, we evaluated the IVF/ICSI outcome in 150 clomiphene-resistant women with PCOS who were treated by ovarian electrosurgical drilling and then compared with 150 patients without history of ovarian drilling. 

Based on our results, the ovarian drilling in patients with PCOS reduces the risk of OHSS, known as a potential life-threatening disorder. PCOS paients respond differently to controlled ovarian hyperstimulation compared with normal ovaries; therefore, they experience a higher cycle cancellation rate due to an exaggerated response to gonadotropin therapy that leading to the increased risk of OHSS (2,6,14). 

Tozer et al. (15) retrospectively compared IVF outcomes between PCOS patients undergoing LOD and PCOS patients who did not undergo LOD. They found a trend toward increased ongoing pregnancy rates and decreased risk of developing severe OHSS despite the fact that all LODtreated patients remained anovulatory after the procedure. 

In another study by Greenblatt and Casper (16), they have showed that ovarian trauma disrupts local androgen synthesis that leads to a reduction in intraovarian androgen concentration that is followed by negative effects of androgen on follicular maturation. Subsequently it results in decreased peripheral conversion of androgen to estrogen that causes positive feedback on LH secretion (17). Although, other factors such as inhibin and other local ovarian substances may be involved (18). 

Breborowicz et al. (19) in their study showed that prior to IVF, transvaginal ovarian drilling in patients with severe PCOS on metformin therapy leads to an increase in E2 level, meaning an increase in number of mature oocytes and embryos as well as available blastocyst. In our paper, the pregnancy rate in the two groups did not differ, but the number of oocytes and embryos obtained was less in patients with history of LOD, although these differences were not clinically significant. A decrease in number of retrieved oocytes and embryos in the current study suggests the possibility of increased risk of diminished ovarian reserve (DOR) or premature ovarian failure. 

Weerakiet et al. (20) in a cross sectional study evaluated the effect of LOD on ovarian reserve. Anti-mullerian hormone (AMH), inhibin B, basal FSH, antral follicle count (AFC) and ovarian volume were measured and compared with related values in PCOS women underwent LOD, PCOS women who did not undergo LOD and normal women with regular menstrual cycles. Their findings revealed that AMH level was lower in LODPCOS group (4.6 ± 3.16 ng/mL) as compared to the non-LOD-PCOS group (5.99 ± 3.36 ng/mL), but the difference was not statistically significant. Furthermore AMH level was significantly lower in normal women with regular menstrual cycles, indicating the reduced risk factors for developing OHSS and good ovarian reserve. The serum FSH mean levels were significantly higher in LOD-PCOS group. There was no significant differences in inhibin B mean levels between groups. Therefore, they concluded that the ovarian reserve was diminished in LOD-PCOS women as compared to non-LOD-PCOS women. 

Mural in his literature review demonstrated that although the available data in the literature is limited, there was no concrete evidence of a diminished ovarian reserve or premature ovarian failure associated with LOD in women with PCOS. He indicated that LOD is considered as an effective method to enhace the overain function and nomalize ovarian morphologic and endocrinologic properties if it is performed properly (21). 

This study has several limitations such as lack of access to early pregnancy outcomes and life birth rate information. However, further studies with bigger samples and a prospective follow-up for a long period of time on electrosurgical drilling effects on ovarian reserve are recommended. Furthermore review of the possibility of premature menopause in LOD-PCOS and its related complication may benefit from further studies. 

## Conclusion

There was no difference in the pregnancy rate in women with clomiphene-resistant PCOS undergoing LOD as compared to patients without history of LOD. Due to significant reduction in OHSS in women undergoing LOD, this surgical treatment may be considered as a useful technique in the management of patients who have previously developed OHSS. Though there are ongoing concerns about long-term effects of LOD on ovarian function. 
